# 775 The Ongoing Impact of the COVID Pandemic on Young Burn Survivors and their Families

**DOI:** 10.1093/jbcr/irac012.328

**Published:** 2022-03-23

**Authors:** Brad Jackson, Kerry Mikolaj, Trudy Boulter, Nichole M Schiffer

**Affiliations:** Children's Hospital Colorado, Aurora, Colorado; Children's Hospital Colorado, Westminster, Colorado; Children's Hospital Colorado, Denver, Colorado; Children’s Hospital Colorado, Denver, Colorado

## Abstract

**Introduction:**

The COVID pandemic continues to bring numerous challenges for young burn survivors and their families. This project addressed the ongoing impact that COVID-19 is still having on youth burn survivors and their caregivers.

Our burn camp program moved to a virtual format for 2020 but returned to an in person camp experience for 2021. This project is an extension of our assessment in 2020 by asking youth and their families to reflect on the persistent effects of COVID-19 into 2021.

**Methods:**

Prior to each camp year, we asked campers (ages 8 – 18) and their caregivers / parents to complete questionnaires about their year, rating and specifying the personal impacts of COVID as part of their overall camp application. We also asked “what has helped you get through tough times this year?” In 2020 we had 47 campers and caregivers / parents participate with an increase to 60 campers and caregivers / parents for 2021.

**Results:**

The majority of youth continued to rate the impact of COVID-19 as “Somewhat” or “Highly” from 2020 to 2021, however the percent of youth rating these higher levels of impact decreased in 2021. The majority of caregivers rated the impact in 2020 as “Somewhat” while the percentage rating these higher levels of impact increased in 2021 with more caregivers also endorsing “Highly”.

Campers AND caregivers / parents identified the same top 3 impacts in 2020 and 2021:

1. Online school / virtual learning

2. Friends / Social

3. Quarantine

The impact on the fourth highest area of Sports / Activities decreased from 2020 to 2021. In both years, youth and caregivers rated Quality time with Family as a positive impact.

Campers and caregivers endorsed Family, Friends, Faith, and What I learned recovering from my burn injury as factors helping them get through tough times.

**Conclusions:**

Children, youth, and families who have experienced a burn injury continue to report both negative and positive impacts from the COVID-19 pandemic.

Not all youth and families are equally affected, but burn survivors and their caregivers rated the highest impacts as online school / virtual learning, friends / social, and quarantine in both 2020 and 2021.

Family and friends were the greatest sources of support during tough times

Burn camp provided the opportunity for connection in the face of ongoing impacts of COVID-19.

